# Identification of low-frequency *TRAF3IP2 *coding variants in psoriatic arthritis patients and functional characterization

**DOI:** 10.1186/ar3807

**Published:** 2012-04-18

**Authors:** Beate Böhm, Harald Burkhardt, Steffen Uebe, Maria Apel, Frank Behrens, André Reis, Ulrike Hüffmeier

**Affiliations:** 1 Divisions of Rheumatology, Department of Internal Medicine II, Johann Wolfgang Goethe University, Theodor-Stern-Kai 7, 60590 Frankfurt am Main, Germany; 2 Institute of Human Genetics, University of Erlangen-Nuremberg, Schwabachanlage 10, 91054 Erlangen, Germany

## Abstract

**Introduction:**

In recent genome-wide association studies for psoriatic arthritis (PsA) and psoriasis vulgaris, common coding variants in the *TRAF3IP2 *gene were identified to contribute to susceptibility to both disease entities. The risk allele of p.Asp10Asn (rs33980500) proved to be most significantly associated and to encode a mutant protein with an almost completely disrupted binding property to TRAF6, supporting its impact as a main disease-causing variant and modulator of IL-17 signaling.

**Methods:**

To identify further variants, exons 2-4 encoding both known TNF-receptor-associated factor (TRAF) binding domains were sequenced in 871 PsA patients. Seven missense variants and one three-base-pair insertion were identified in 0.06% to 1.02% of alleles. Five of these variants were also present in 931 control individuals at comparable frequency. Constructs containing full-length wild-type or mutant *TRAF3IP2 *were generated and used to analyze functionally all variants for TRAF6-binding in a mammalian two-hybrid assay.

**Results:**

None of the newly found alleles, though, encoded proteins with different binding properties to TRAF6, or to the cytoplasmic tail of the IL-17-receptor α-chain, suggesting that they do not contribute to susceptibility.

**Conclusions:**

Thus, the *TRAF3IP2*-variant p.Asp10Asn is the only susceptibility allele with functional impact on TRAF6 binding, at least in the German population.

## Introduction

**Figure 1:**
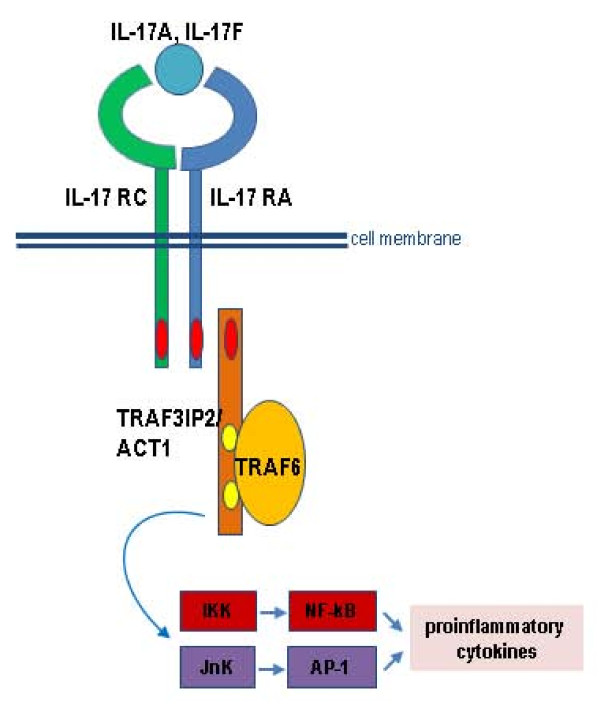
**Interleukin (IL)-17A/IL17F binds to the heterodimeric IL-17R and leads to its signaling**. Signaling includes recruitment of nuclear factor-κB activator 1 (ACT1) via SEFIR domains (red ovals). Activation of ACT 1 encoded by *TRAF3IP2 *allows binding to TRAF6 via TNF receptor-associated factor (TRAF)-binding domains (yellow ovals); this activates NF-κB as well as proinflammatory cytokines.

Recent genome-wide association studies (GWASs) for psoriatic arthritis and psoriasis vulgaris have been quite successful and revealed more than 25 new susceptibility loci [1-7] characterizing psoriatic arthritis (PsA), mainly as an immune-mediated disease that is also affected by barrier proteins. One new PsA and psoriasis-susceptibility gene, *TRAF3IP2*, codes for the adaptor protein ACT1 (nuclear factor-κB activator 1), a regulator of the NF-κB pathway involved in IL-17 signaling. We were able to identify the common coding variant p.Asp10Aspn (rs33980500) as the disease-causing allele with evidence from association findings at single nucleotide polymorphism (SNP) and haplotype levels, as well as binding studies of ACT1 with its interaction partner TRAF6 [2] (Figure 1). However, despite the exponentially increased knowledge of PsA and psoriasis susceptibility, the sum of the currently identified genetic risk factors does not explain the entire genetic contribution to disease.

At the common susceptibility gene *IL23R*, coding for interleukin-23 receptor for Crohn disease, low-frequency variants have been identified as protective factors, and their effects turned out to be larger than those of common variants and accounted for a greater portion of genetic contribution [8]. To find out whether low-frequency coding variants in *TRAF3IP2 *contribute to PsA susceptibility, we sequenced exons encoding TRAF-binding domains (Figure 1) [9] and studied their association with PsA as well as the functional consequences of the respective amino acid changes on interaction with TRAF6 and the interleukin-17 receptor α (IL17-RA).

## Materials and methods

### Study population

We analyzed a case-control study comprising 871 PsA patients. A subset of 764 patients was described previously [2]. All patients fulfilled the recently defined CASPAR (for ClASsification of Psoriatic ARthritis) criteria [10] and were recruited between 2002 and 2011 exclusively by board-certified rheumatologists at five different rheumatology departments in Germany. In comparison to the previously described cohort, clinical characteristics were very similar: the mean (SD) age of onset for PsV was 28.3 ± 13.3 years; 60.8% of the patients were men. For 78% of the patients, the diagnosis of PsA was made ≥ 3 years before recruitment, and 96% of patients had a skin involvement ≥ 3 years before study inclusion. Peripheral joint involvement was detectable in the majority of cases (827 or 94.8%); this was oligoarticular in 183 patients and polyarticular in 644 (21.0% and 73.8% of the entire cohort, respectively). Diagnosis of spinal involvement was based on symptoms of inflammatory back pain, characteristic clinical signs of restricted vertebral movement and/or sacroiliac pain on physical examination, and a subsequent confirmation by radiographic signs of sacroiliitis or spondylitis or both. Spinal involvement was observed in 204 patients, accounting for 23.4% of the PsA cohort. In these patients, sacroiliitis or spondylitis or both were partly associated with concomitant peripheral joint disease.

The 931 German control individuals were healthy blood donors and the same as previously reported [2].

Ethical approvals for all German studies were obtained from the Ethics Committees of the medical faculties of the Universities of Erlangen, Münster, and Frankfurt/Main. All probands gave written informed consent. The investigations were conducted according to the Declaration of Helsinki principles.

### Sequencing

All PsA patients were sequenced for exons 2-4 coding for TRAF-binding domains. Mainly intron-based primers for four amplicons were designed with Primer 3 (v.0.4.0) [11]. In general, Sanger sequencing was performed, as described before [12], with the following changes: thermocyclers were Thermo Multiblock (ThermoFisher Scientific, Ulm, Germany); the robotic system for PCR (polymerase chain reaction) clean-up as well as for sequencing reactions was Beckman-Coulter Biomek Nxp (kit for PCR clean-up: Agencourt AMPure; kit for sequencing reactions: CleanSEQ; Beckman-Coulter, Krefeld, Germany). Sequences were analyzed with the Software Sequencher versus 4.10.1 (Gene Codes, Ann Arbor, MI, USA). Genotyping rates of the four amplicons were between 99.8% and 100%. Variants were named according to the usual naming conventions with regard to *TRAF3IP2 *reference sequence NM_147686 (coding for 565 amino acids).

### Analysis of variants in controls

Control individuals were genotyped for all missense variants as well as the 3bp-insertion with self-designed TaqMan assays (Life Technologies, Carlsbad, CA, USA), as previously described [2]. Missing genotypes or carriers of low-frequency variants were resequenced, resulting in a genotyping rate of 100% for seven variants and 99.8% for the further one.

### Statistics

Fisher Exact test, as implemented in R (version 2.13.1) [13], was used for comparison of frequency distributions between cases and controls.

### Protein-binding analyses in mammalian two-hybrid system

A mammalian two-hybrid assay (Stratagene) was applied to analyze the interaction of wild-type and mutant protein ACT1 of *TRAF3IP2 *with TRAF6 and IL-17RA, as described before [2]. As a change to previous analyses, full-length *TRAF3IP2 *mRNA (coding for amino acid residues 1 through 565) was amplified from reverse-transcribed cDNA of the chondrocyte cell line T/C28a4 by using 5'-TGAATTCATGAACCGAAGCATTCCTGTG-3' and 5'-TGCGGCCGCTCACAAGGGAACCACCTGAAG-3'. The cytoplasmic tail of IL-17RA (amino acid residues 343-667, NM_014339) was amplified from reverse-transcribed cDNA of the human macrophage cell line THP-1 by using 5'-AGGATCCATGACCTGGAGGCTAGCTGGGCCTGGAA-3' and 5'-AGCGGCCGCTCAGGTGTGGAGGGGCTGCGGCGCTGGCTGA-3', and cloned into the *Bam*HI/*Not*I site of the pCMV-BD bait vector. HEK 293 cells (1 × 10^4^) were grown in 96-well white tissue-culture plates (Greiner) for 24 hours, and the bait and prey vectors (25 ng each) were co-transfected with a firefly luciferase reporter (250 ng) by using jetPEI reagents according to the supplier's instructions (PeqLab). Transfection efficiency was controlled by co-transfecting a renilla luciferase plasmid (5 ng, pRL-TK vector; Promega). At 48 hours after transfection, luciferase activity of quadruplicate wells was measured by using the Dual Glo Assay System (Promega) on a Mithras LB940 plate reader (Berthold Technologies). Assays were performed ≥ 5 times.

For functional testing of the two common as well as the newly identified rare coding variants of *TRAF3IP2 *[2], constructs were generated by site-directed mutagenesis by using the QuickChange site-directed mutagenesis kit (Stratagene). Quality control of all vectors was performed with DNA sequencing. The firefly luciferase values were normalized to the Renilla values. Statistical significance was determined by using an unpaired Student *t *test.

## Results

Sanger sequencing in 871 PsA patients revealed eight low-frequency variants: one 3-bp insertion and seven missense variants, one of them being the known SNP rs61756667 (minor allele frequency of 0.10% in the database), as indicated in Table 1. Allele frequencies ranged between 0.06% and 1.03% in PsA cases and 0 to 0.54% in control individuals, respectively. Distributions of allele frequency for single low-frequency variants as well as the sum of the eight variants (*P *= 0.493) did not show significant differences between cases and controls.

**Table 1:** Association analysis of new variants

Coding position	Protein position	Position hg19	Allele	871 PsA patients		931 control individuals		*P *(Fisher Exact)
				***n ***	**%**	***n ***	**%**	

c.105_106 InsACC	p.Pro35dup	111913185	Ins	2	0.11	3	0.16	1
			WT	1,740	99.89	1,859	99.84	
c.281G > A (rs61756667)	p.Ser94Asn	111913009	A	1	0.06	4	0.21	0.376
			G	1,737	99.94	1,858	99.79	
c.350C > T	p.Ala117Val	111912940	T	1	0.06	0	0	0.483
			C	1,737	99.94	1,858	100.00	
c.649C > A	p.Pro217Thr	111912641	A	18	1.03	10	0.54	0.127
			C	1,724	98.97	1,852	99.46	
c.691C > T	p.Leu231Phe	111912599	T	2	0.11	1	0.05	0.613
			C	1,740	99.89	1,861	99.95	
c.746A > G	p.Gln249Arg	111912544	G	1	0.06	0	0	0.483
			A	1,741	99.94	1,862	100.00	
c.1058G > C	p.Gly353Ala	111896989	C	1	0.06	0	0	0.483
			G	1,741	99.94	1,862	100.00	
c.1184A > G	p.Asn395Ser	111896863	G	3	0.17	7	0.38	0.346
			A	1,739	99.83	1,855	99.62	

WT, wild type. Names, position on chromosome 6, and count/frequency of wild-type alleles/low-frequency alleles of new variants in PsA patients and control individuals are shown, as well as the *P *value of Fisher Exact test.

**Figure 2:**
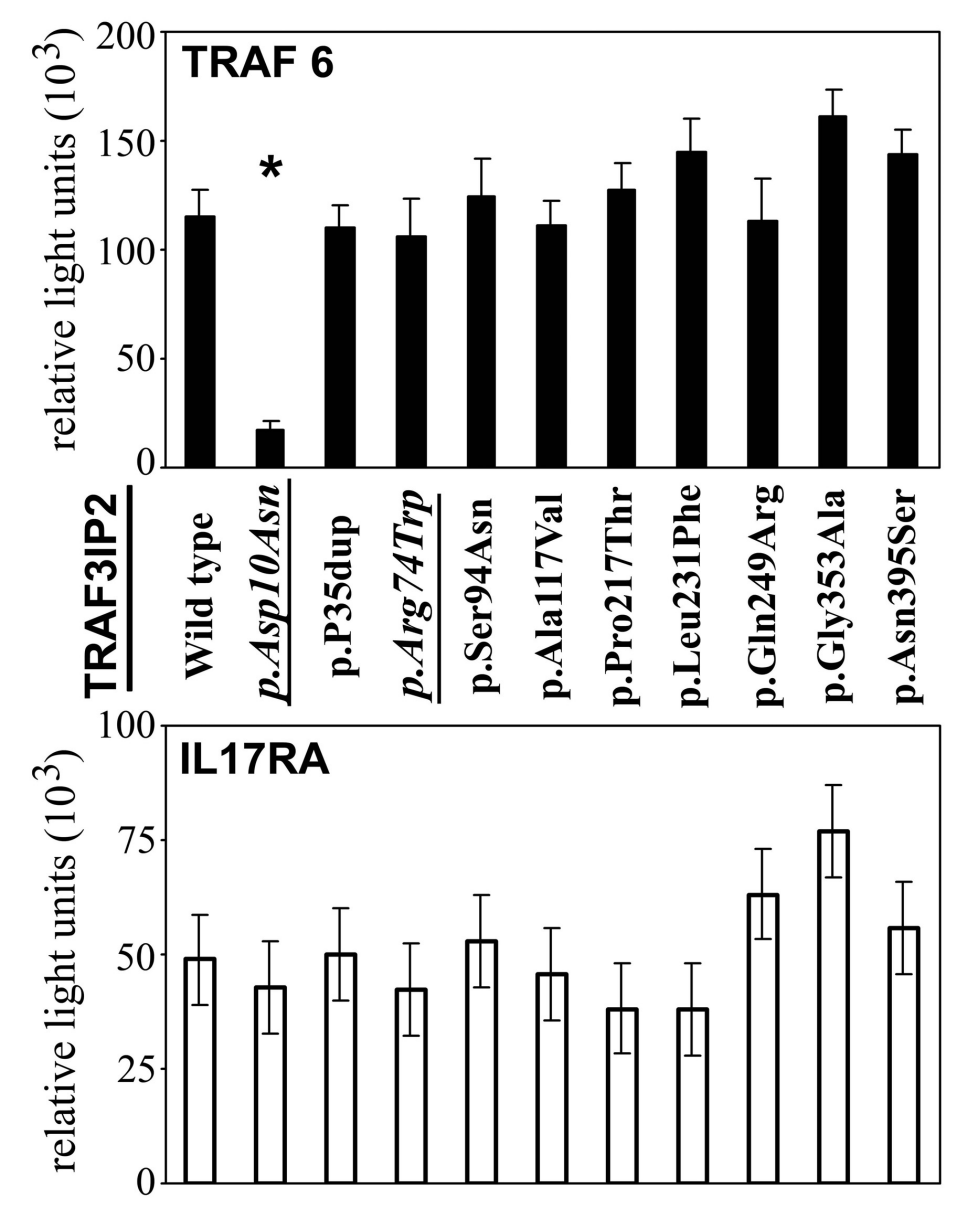
**Binding of ACT1 and its mutant variants to TNF receptor-associated factor (TRAF)6 and interleukin (IL)-17RA**. Interaction of wild-type *TRAF3IP2*/ACT1 and its mutant variants with either TRAF6, upper panel, or IL-17RA, lower panel, was determined by mammalian two-hybrid system. The *TRAF3IP2 *mutant p.Asp10Asn showed a significantly reduced binding to TRAF6. Common coding variants indicated by underlined and italicized letters. *P < 0.005, when compared with wild-type *TRAF3IP2*.

Because our case-control study of ~900 individuals was of limited power to exclude association to very-low-frequency variants (for example, found on one allele of one patient), we investigated the impact of amino acid changes encoded by the new *TRAF3IP2-*variants on functional properties of the ACT1 protein also by mammalian two-hybrid technology. None of the amino acid mutations in the full-length constructs of the new *TRAF3IP2 *coding variants exhibited any influence on TRAF6 binding (Figure 2). Also, TRAF6 interaction of the common coding variant rs13190932 (p.Arg74Trp) was comparable to wild-type, in agreement with earlier results obtained in assays using shortened *TRAF3IP2 *constructs [2]. In contrast, the analysis of a full-length *TRAF3IP2 *construct, containing the earlier described disease-causing variant rs33980500 (p.Asp10Asn), revealed a nearly completely disrupted interaction with TRAF6 (~90%), thereby confirming previous analyses. Moreover, the ability to interact with the cytoplasmic IL-17RA domain did not differ between ACT1 proteins encoded by either wild-type *TRAF3IP2 *or by any of its low-frequency variants. In this respect, p.Asp10Asn did not cause different results compared with other *TRAF3IP2 *variants.

## Discussion

Our in-depth sequencing of TRAF-binding domains in *TRAF3IP2 *revealed a number of new low-frequency variants in PsA patients occurring at a similar frequency in control individuals. This finding is not unexpected, because, for example, newly identified low-frequency variants in most of the susceptibility genes identified in GWAS and sequenced for Crohn disease [8] or type I diabetes [14] were not associated with disease. Further evidence that our newly identified coding variants do not cause disease was derived from functional analyses: none of the variants leading to amino acid changes in the respective ACT1 proteins affected TRAF6 interaction. Reassuringly, we could confirm previous results of a dramatically reduced TRAF6 binding for the ACT1 variant harboring the disease-causing variant p.Asp10Asn under assay conditions analyzing full-length *TRAF3IP2-*transcripts (Figure 2). Binding of ACT1 to the cytoplasmic domain of IL-17RA is mediated via a C-terminal SEFIR domain that remained conserved in wild-type configuration in all investigated *TRAF3IP2 *constructs (Figures 1 and 2). Accordingly, a rather limited *a priori *likelihood existed to detect major differences in IL-17RA-binding properties of the investigated mutant *TRAF3IP2 *constructs, although an indirect modulatory effect of conformational changes induced by the distantly located mutations in the TRAF-binding domains could not be entirely excluded. However, our studies did not reveal an altered IL-17RA interaction for any of the nine investigated coding variants.

## Conclusions

Our data indicate that the common variant rs33980500 (p.Asp10Aspn) is the only risk allele for PsA with functional impact on TRAF6 binding, at least in the German population.

## Abbreviations

ACT1: nuclear factor-κB activator 1; CASPAR: ClASsification of Psoriatic Arthritis; GWAS: genome-wide association studies; IL-17RA: interleukin-17 receptor A; NF-κB: nuclear factor-κB; PCR: polymerase chain reaction; PsA: psoriatic arthritis; PsV: psoriasis vulgaris; SNP: single-nucleotide polymorphism; TRAF: TNF receptor-associated factor; TRAF3: TNF receptor-associated factor 3; TRAF6: TNF receptor-associated factor 6; TRAF3IP2: TRAF3-interacting protein 2.

## Competing interests

The authors declare that they have no competing interests.

## Authors' contributions

HB, AR, and UH designed the study and worked out its concept. UH planned and performed the genotyping, and SU and UH analyzed genetic data. BB and HB performed functional studies. BB, HB, MA, FB, and UH recruited subjects and collected phenotypic data. UH, BB, and HB wrote a first draft of the manuscript; all authors reviewed and approved the manuscript.
